# Assessing Spatial Flood Vulnerability at Kalapara Upazila in Bangladesh Using an Analytic Hierarchy Process

**DOI:** 10.3390/s19061302

**Published:** 2019-03-15

**Authors:** Muhammad Al-Amin Hoque, Saima Tasfia, Naser Ahmed, Biswajeet Pradhan

**Affiliations:** 1 Centre for Advanced Modelling and Geospatial Information Systems (CAMGIS), Faculty of Engineering and IT, University of Technology Sydney, Ultimo, NSW 2007, Australia; MuhammadAl-Amin.Hoque@uts.edu.au; 2 Department of Geography and Environment, Jagannath University, Dhaka 1100, Bangladesh; saimatasfia28@gmail.com (S.T.); naserbipu.geo2011@gmail.com (N.A.); 3 Department of Energy and Mineral Resources Engineering, Choongmu-gwan, Sejong University, 209 Neungdong-ro, Gwangjin-gu, Seoul 05006, Korea

**Keywords:** floods, vulnerability, remote sensing, GIS, analytical hierarchy process, Bangladesh

## Abstract

Floods are common natural disasters worldwide, frequently causing loss of lives and huge economic and environmental damages. A spatial vulnerability mapping approach incorporating multi-criteria at the local scale is essential for deriving detailed vulnerability information for supporting flood mitigation strategies. This study developed a spatial multi-criteria-integrated approach of flood vulnerability mapping by using geospatial techniques at the local scale. The developed approach was applied on Kalapara Upazila in Bangladesh. This study incorporated 16 relevant criteria under three vulnerability components: physical vulnerability, social vulnerability and coping capacity. Criteria were converted into spatial layers, weighted and standardised to support the analytic hierarchy process. Individual vulnerability component maps were created using a weighted overlay technique, and then final vulnerability maps were produced from them. The spatial extents and levels of vulnerability were successfully identified from the produced maps. Results showed that the areas located within the eastern and south-western portions of the study area are highly vulnerable to floods due to low elevation, closeness to the active channel and more social components than other parts. However, with the integrated coping capacity, western and south-western parts are highly vulnerable because the eastern part demonstrated particularly high coping capacity compared with other parts. The approach provided was validated by qualitative judgement acquired from the field. The findings suggested the capability of this approach to assess the spatial vulnerability of flood effects in flood-affected areas for developing effective mitigation plans and strategies.

## 1. Introduction

Floods are regarded as among the most devastating hydro-meteorological natural disasters. These disasters often cause tremendous economic and environmental damages and loss of lives [1,2,3]. The United Nations (UN) report states that approximately 2.3 billion people were affected and 157,000 died by floods in 1995–2015 worldwide [4]. Globally, floods cause nearly US 386 billion dollar economic loss in the last three decades of the twentieth century [1]. Several recent studies have predicted and expected that the occurrence rate and intensity of flood disasters are likely to be considerably increased under future climate change scenarios [5,6,7,8]. Moreover, other factors, such as rapid urbanisation, population growth and economic development, will intensify the flood risk areas worldwide [3]. As a result, people, properties and the environment will be under constant risk in the future.

Prevention and reduction are appropriate strategies in disaster management for reducing the effects of flood disasters [9]. Deriving spatial information regarding the key vulnerable infrastructure and areas, the level of vulnerability and the factors liable for vulnerability is necessary to develop suitable flood mitigation options [10,11]. Spatial vulnerability assessment can provide the aforementioned information in detail. The spatial vulnerability is to map the extent to which people, property, resources, and environment are likely to be affected by a hazard [12,13,14]. The effective spatial vulnerability assessment includes mapping of various criteria that influence different types of vulnerability and coping capacity and their integration to obtain the actual vulnerability scenario [15,16]. The maps produced by vulnerability assessment could be used by policymakers for effective management plans targeting prevention and reduction measures [17,18,19]. Thus, the vulnerability assessment can contribute to the mitigation of the effects of floods on people, property and the environment.

Several studies have been performed using geospatial techniques for mapping vulnerability though various approaches [6,19,20,21,22,23]. Appropriate and sufficient criterion selection, scale and components of vulnerability determine the detailed and accurate vulnerability information [13]. The reliability of vulnerability information is enhanced by selecting adequate criteria of each vulnerability component (e.g., physical and social vulnerabilities and coping capacity) and their standard processing [15]. In addition, other factors, such as study area scale (local or regional), influence the derivation of detailed vulnerability information [1,24,25]. Detailed and accurate vulnerable information helps prepare the optimal flood mitigation plans [24]. However, a comprehensive flood vulnerability model is rare in literature because most current studies have been performed on the basis of particularly limited criteria and at the regional scale [18,20,22,26]. Selection of appropriate components is another major issue in vulnerability analysis. Coping capacity of the local community, surrounding environments and resources have an important role to protect and minimise flood effects [15]. Therefore, integrating coping capacity for assessing vulnerability to derive actual result is essential [27]. Few studies are found in the current literature where coping capacity is adopted in the spatial vulnerability analysis using multi-criteria-integrated geospatial techniques at the local scale [15,28].

Bangladesh is a country highly affected by floods [20]. Every year various parts of the country are affected [29]. However, studies related to detailed flood vulnerability assessment using geospatial techniques in Bangladesh are very limited. A few studies are found in the literature [15,19,20,26,30], but most of them have considered very few criteria for assessing flood vulnerability without integrating all of the components of vulnerability. Masood and Takeuchi [30] used house/living place and land covers in their flood vulnerability assessment in mid-eastern Dhaka city, whereas Bhuiyan and Baky [20] used only land use as a criterion for flood vulnerability assessment in Sirajganj Sadar Upazila. Topography and land cover data were considered by Bhuiyan and Dutta [26] in the flood vulnerability assessment study in south-western region of Bangladesh. On the other hand, several social and coping capacity criteria were considered by Roy and Blaschke [19] in spatial flood vulnerability assessment in Dacope Upazila, Khulna. Dewan [15] also assessed urban flood vulnerability integrating several criteria in Dhaka city.

Geospatial approach integrating remote sensing and spatial analysis are highly effective techniques for obtaining spatial flood vulnerability information [1,3,7]. Remote sensing supports the capability to provide repeated satellite imagery for deriving spatial environmental data where spatial analysis helps in the collection, analysis and integration of various datasets for spatial decision-making [31]. Weighting and ranking are required in the spatial decision-making processes to incorporate multi-criteria for assessing spatial vulnerability. Analytic hierarchy process (AHP) is considered an optimal method for integrating multi-criteria for special decision-making to generate spatial vulnerable information [1,2,19]. Multi-criteria layers are analysed in the AHP environment for developing a hierarchical structure that provides weighting and ranking with the guidance of experts and users [2,32].

This study aims to develop and examine a multi-criteria-integrated approach of spatial vulnerability mapping to assess flood effects using AHP incorporating information produced from spatial analysis integrating GIS and statistical analysis, optical remote sensing and field data on coping capacity as well as validation data. The specific objectives of this study are as follows: (1) to develop a spatial vulnerability mapping approach that integrates multi-criteria for flood effects at the local scale covering <1000 km^2^; (2) to examine the developed approach for assessing spatial flood vulnerability at the local scale in Kalapara Upazila, Bangladesh; and (3) to evaluate the validation of spatial vulnerability assessment approach.

## 2. Materials and Methods

### 2.1. Study Area

**Figure 1:**
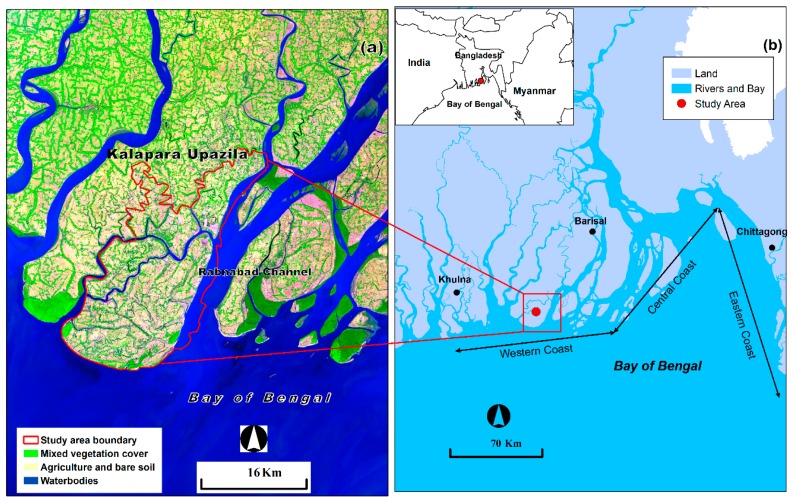
(**a**) Location of study area mapped on Landsat 8 OLI (Operational Land Imager) image of 20 January 2018, (**b**) study area in the context of the coastal region of Bangladesh.

This research was performed in Kalapara Upazila, a local administrative region (approximately 483.08 km^2^) of Patuakhali district in the coastal area of Bangladesh (Figure 1). Kalapara Upazila is located between 21°48′ to 22°05′ north latitudes and 90°05′ to 90°20′ east longitudes. The area is surrounded by several large coastal rivers, such as Andharmanik, Nilganj and Dhankhali, and the Rabnabad channel and is open to the ocean in the southern side. This coastal area falls within a tropical climate. The average annual temperature is 25.9 °C. Substantial rainfall is experienced most months of the year. The average annual rainfall is 2654 mm. Bangladesh is considered a highly flood-affected country [20]. Flood is a frequent event in the study area [29]. Human and all types of resources are highly vulnerable to flooding effects due to the area’s lowland, geographical location and dense population [6]. The economic condition of the people in the study area is poor. Numerous people live under the poverty line [33]. Therefore, the effects of floods considerably affect the socio-economic conditions. Frequent cyclone-induced storm surges, intensive rainfall and degradation of water storing wetlands are triggering points for floods in this area.

### 2.2. Method Overview

In this paper, an AHP-based geospatial multi-criteria assessment technique was adopted to combine various natural, social and anthropogenic criteria for flood vulnerability assessment. Several criteria can be easily integrated and aggregated and present output in a particularly simple manner [17,34]. Few vulnerability equations are available for the vulnerability mapping of any hazard (natural or manmade) [32]. An advanced and complete equation can provide an effective vulnerability assessment. Equation (1) is selected in this study for flood vulnerability assessment in accordance with the review of existing literature [13,15]:

(1) Vulnerability=Physical vulnerability×social vulnerability/coping capacity

**Figure 2:**
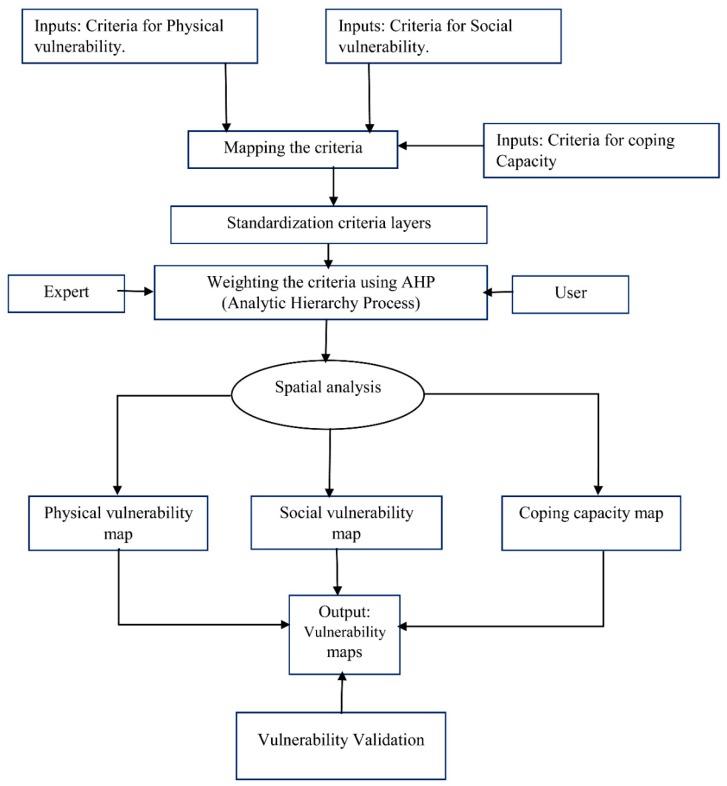
Methodological flowchart of the vulnerability assessment approach followed in this study.

Figure 2 outlines the methodological flowchart followed in the current study.

### 2.3. Datasets and Sources

Dynamic criteria were selected for vulnerability assessment in the present study. We used a wide range of data from various sources for creating spatial criterion layers using geospatial techniques. We collected these data from national and international institutions and fieldwork. Validation and coping capacity data were acquired through the fieldwork conducted in October 2018 in the study area. The coping capacity data covered flood shelter and health complexes. Table 1 details the characteristics of datasets used in the current study.

### 2.4. Vulnerability Evaluation Criteria, Alternatives and Mapping

The criteria and alternatives were selected on the basis of the literature, availability of data and their relevance and influence on flood vulnerability in the present study. The spatial thematic layers of each selected criterion were generated by mapping the alternatives of each criterion. We produced 16 spatial thematic layers under three vulnerability components in this study. The spatial resolution was set to 30 m × 30 m cell size for each raster layers. Numerous spatial criterion layers were processed and prepared using ArcGIS software (version 10.4). The relative importance and mapping procedures of the selected criteria are described in the subsequent sections.

#### 2.4.1. Criteria for Physical Vulnerability Mapping

Vulnerability is controlled and influenced by physical/natural factors. These controlling factors have been selected as criteria for this analysis. In this study, five physical vulnerability criteria (i.e., land use and cover, distance to the active channel, slope, elevation and precipitation intensity) were selected for vulnerability assessment [15,18,19,35].

**Figure 3:**
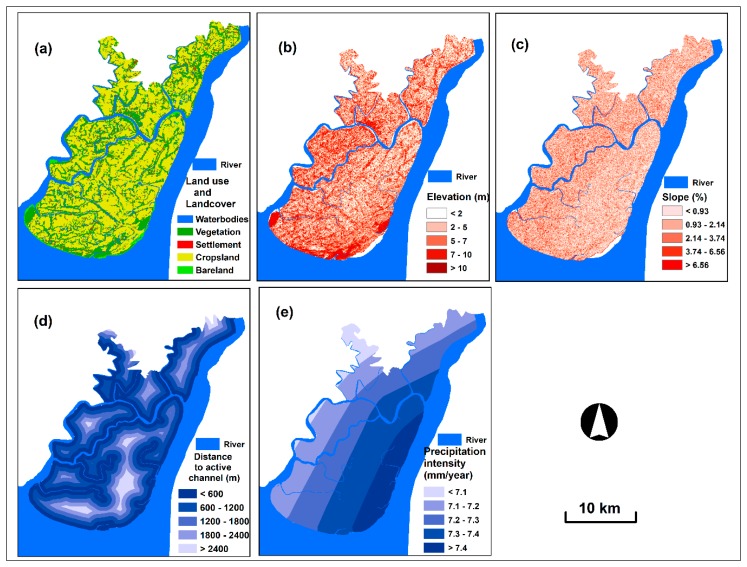
Physical vulnerability criterion layers: (**a**) land use and cover, (**b**) elevation, (**c**) slope, (**d**) distance to the active channel and (**e**) precipitation intensity.

Damage and effects of floods are high for certain types of land covers. We used the Landsat OLI imagery to map land use and cover (Figure 3a). A hybrid classification scheme was applied to classify six land use and cover categories, namely, river channel, open water bodies, vegetation, settlement and crops and bare lands. Firstly, unsupervised clustering algorithm was conducted to identify the potential classes and then training sample data were selected and used to implement supervised classification using maximum likelihood algorithm [36]. We used the ENVI 5.4 to pre-process the image and ERDAS IMAGINE 2017 for hybrid classification. Accuracy assessment of the produced map was conducted by collecting 250 random points from high spatial resolution Google Earth imagery (2017) of the study area. Stratified random sampling technique was used to acquire the reference points with minimum 50 points for each cover class. The study followed the techniques described in [37,38] to perform the accuracy assessment. The overall accuracy of the produced map was 90%.

The elevation and slope have a great influence on spatial flood vulnerability assessment. The low and plain areas with gentle slope are more vulnerable to flood than those with high elevation and steep slope [39]. The elevation and slope spatial criterion layers were produced from the modified Shuttle Radar Topography Mission (SRTM) digital elevation model (DEM) at 30 m resolution (Figure 3b,c). Tree offsets are present in the SRTM data as radar used for the SRTM mission cannot penetrate the tree canopies fully [31,40]. These tree offsets were removed using a tree height offsets estimation method provided by Gallant, et al. [41]. Void filled areas and spike were also removed from DEM before use in this study converting into points and re-interpolated [31]. Distance to the active channel is a conditioning factor in the level of flood vulnerability for any region. In general, the area close to the active channel is more vulnerable to floods than that far from the channel [30]. In this study, river channel data are used for generating distance to active channel map (Figure 3d).

Precipitation intensity is a highly important criterion that extensively influences flood vulnerability [18]. The areas with high compared with low precipitation intensity are more vulnerable to floods. Precipitation intensity map was prepared using the daily precipitation data (1950–2017) acquired from BMD. In this process, we initially created a map of annual precipitation by interpolating 35 rainfall stations of Bangladesh (Figure 3e). We applied the kriging interpolation technique using ArcGIS software for this process. Kriging interpolation is a widely used technique for interpolating precipitation data. This technique is unbiased and has minimum variances [42]. Then, we extracted the study area from this map.

#### 2.4.2. Criteria for Social Vulnerability Mapping

The incapability of people, organisations and societies to cope with the adverse effects of hazards for their social interactions, institutions and systems of cultural values is referred to as social vulnerability [43]. Several social criteria influence social vulnerability to floods. These criteria were selected here for mapping social vulnerability. A total of eight social criteria, namely, population density, dependent population, disabled population, female population, wooden house, households with ponds and others, households with no sanitation and agriculture-dependent population, were selected.

**Figure 4:**
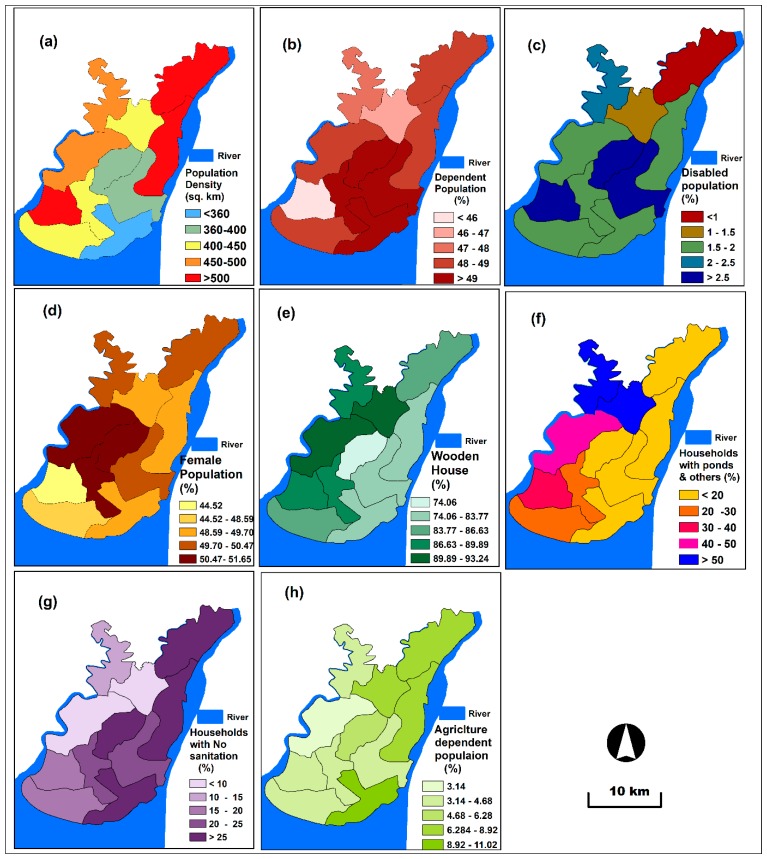
Social vulnerability criterion layers: (**a**) population density, (**b**) dependent population, (**c**) disabled population, (**d**) female population, (**e**) wooden house, (**f**) households with ponds and others, (**g**) households with no sanitation, (**h**) agriculture-dependent population.

Population density is an essential criterion for determining social vulnerability given that people are physically and psychologically affected by flood in several cases [15,43]. Moreover, conducting evacuation activities during and after a flood event is particularly challenging. The population density layer was created using 2011 population census data (Figure 4a). The population census is conducted in Bangladesh within 10 years’ interval and the next census is scheduled to conduct in 2021. These census data were acquired from the BBS. The area with high population density is expected to be more vulnerable than that with low population density.

People less than 15 years and more than 60 years are considered dependent because they do not earn money and depend on other family members. Dependent population, for example, children and elderly people, is highly vulnerable to flood due to their limited mobility and difficulties in emergency evacuation activities [35]. In the present study, the spatial thematic layer for the dependent population was produced using 2011 population census data (Figure 4b).

Floods physically and psychologically affect the disabled population. Disabled people cannot proceed to flood shelters as fast as the active population. Consequently, disabled people are highly affected by floods. Disabled people were extracted and then classified into five categories: ages <1, 1–1.5, 1.5–2, 2–2.5 and >2.5. Females are also affected by floods in numerous ways due to their limited mobility and difficulty with evacuation during emergency cases [44]. The female population is more vulnerable than the male population. Females who are affected by floods are categorised into five classes. Disabled and female population data were collected from the 2011 population census prepared by the BBS to generate disable and female population spatial layers (Figure 4c,d).

Flood vulnerability is also largely influenced by housing quality for a given area. A wooden house is one of the dominant housing types in the study area. Wooden houses are categorised into five groups for preparing spatial layer in this study (Figure 4e). By contrast, water source and sanitation of a particular area are important criteria for assessing social vulnerability to floods. People greatly suffer and are affected by various waterborne diseases due to inadequate access to safe water and hygienic sanitation system. The pond is a dominant water source in the study area. Therefore, households with ponds are divided into five categories and mapped (Figure 4f). Similarly, households with sanitation system are grouped into five classes and mapped to convert into spatial thematic layer (Figure 4g). Agricultural crops are extensively devastated by floods. In this study, the agriculture-dependent spatial layer was prepared by categorising the percentages of dependency into five classes (Figure 4h). The data for the wooden house, household with ponds, household with sanitation and agricultural-dependent population were extracted from the 2011 population census.

#### 2.4.3. Criteria for Coping Capacity Mapping

Coping capacity refers to the capability of people, organisations and systems to manage the effects of disasters using available skills and resources [28]. This component helps in mitigating disaster effects [45]. Three coping capacity criteria, namely, distance to flood shelter, distance to health complex and literacy rate, were selected in this study.

**Figure 5:**
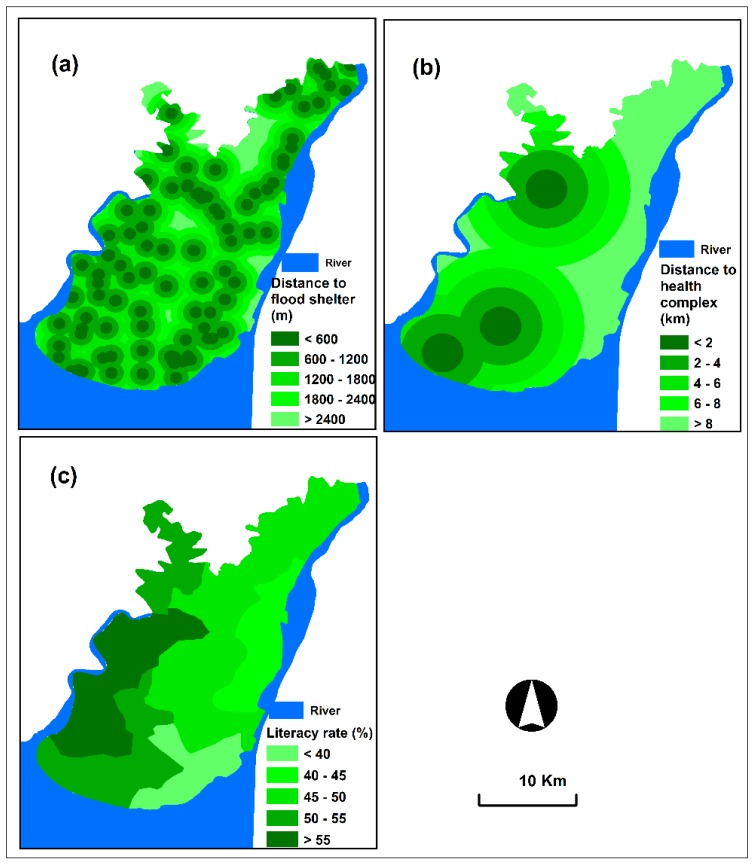
Coping capacity criterion layers: (**a**) distance to flood shelter, (**b**) distance to health complex and (**c**) literacy rate.

The availability of flood shelters and health complexes and their closeness to individual living places are important criteria for assessing the coping capacity of communities [15]. Immediate access capacity to flood shelters and health complexes of every affected individual can largely decrease disaster effects. In this study, we used a global positioning system device to collect spatial flood shelter and health complex data directly from the field. Then, spatial layers, such as distance to flood shelters and health complexes, were created using the ‘Euclidean distance’ technique in the ArcGIS platform (Figure 5a,b).

Literature is an essential criterion that helps people reach appropriate decisions and engage in effective mitigation measures for addressing or recovering from disaster effects [15]. Studies prove that households with literate people exhibit high coping capacity with disaster effects compared with households with illiterate people [46]. Literacy rate data were extracted from the 2011 population census, and a spatial layer was produced in the ArcGIS environment (Figure 5c).

### 2.5. Alternative Ranking and Standardisation Criterion Layer

Ranking was performed on the mapped alternatives of each spatial criterion layer, thereby providing the vulnerability levels (1 to 5) (Table 2). Ranks 1 and 5 indicate very low and high vulnerabilities, respectively. Ranking of alternatives was conducted in accordance with the contribution of vulnerability and AHP guidelines. All spatial layers were transformed into 30 m pixel raster ones to apply the raster-based weighted overlay procedure. Afterwards, standardisation was performed on the alternatives of each spatial criterion layer to convert their ranked values into a common scale (0 to 1) to support the multi-criteria decision using the AHP. Linear scale transformation Equation (2) was applied for this standardisation:

(2) $$p=\frac{x-\min}{\max-\min},$$

where *p* means standardised score; min and max indicate the minimum and maximum values of each dataset, respectively; and x presents the cell value.

### 2.6. Weighting the Criteria Using AHP

In this study, we used the AHP technique for weighting the criteria of physical and social vulnerabilities and coping capacity. The pairwise comparison matrices were developed to weight the criteria using the qualitative judgment received from five experts and a user. The criteria were weighted in accordance with the scale of relative importance proposed by Saaty [47] (Table 3). The expert selection was at the national level in accordance with the related research experiences and in-depth knowledge of the experts and users. The experts and users came from academic, governmental and research organisations. The total score of physical and social vulnerabilities and coping capacity was 1.

The consistency ratio (CR) was computed to check the consistency of comparisons in the pairwise comparison matrix. CR is considered at the acceptable level if the value is equal to or less than 0.1 [48]. Otherwise, a review of the provided qualitative judgement and recalculation of weights is required. The following equation was used to calculate the CR:

(3) CR=Consistency Index/Random Index,

where random index (RI) denotes the randomly generated average consistency index and consistency index (CI) is defined as follows:

(4) $$\mathrm{CI}=(\lambda_{max}-n)/\left(n-1\right),$$

where λ_max_ represents the largest eigenvalue of the matrix and *n* refers to the order of the matrix [49].

Table 4 presents the criterion weights produced from the pairwise comparison matrices and CR values of comparisons.

### 2.7. Vulnerability Assessment

We separately applied the weighted overlay technique with physical and social vulnerabilities and coping capacity spatial criterion layers by incorporating their related criterion weights. Accordingly, we obtain the indices of physical and social vulnerabilities and coping capacity. Then, we categorised the particular index values into five classes (i.e., very low, low, moderate, high and very high) to create the maps of physical and social vulnerabilities and coping capacity. Afterwards, a vulnerability without coping capacity index was created by multiplying the physical and social vulnerability indices. By contrast, a vulnerability integrated coping capacity index was created by multiplying the physical and social vulnerability indices and then dividing them using the coping capacity index in the ArcGIS environment on the basis of Equation (1). Subsequently, we standardised both vulnerability index values on the basis of Equation (2) in the scale of zero to one and categorised them into five levels of vulnerability, namely, very low, low, moderate, high and very high. The natural break statistical method was used to classify flood vulnerability maps. This is because this classification method was found more consistent and efficient to present the spatial pattern of flood vulnerabilities in the study area [50,51].

### 2.8. Validation of Vulnerability Assessment

No established specific method can validate spatial vulnerability mapping approach. However, a qualitative validation method was adopted to evaluate the spatial vulnerability maps [19]. A field visit was performed in October 2018 to assess the accuracy of our software-generated vulnerability maps. The field visit included in-depth personal observation and discussion with around 60 people consisting of local people, experts and policymakers for their opinion regarding the accuracy of the produced spatial flood vulnerability maps. Personal observation involved the identification of specific vulnerable areas from the generated maps, and the area was visited to justify real vulnerability to flood effects. The previous historical flood effects were also explored through a discussion with the local people.

## 3. Results and Discussion

### 3.1. Physical Vulnerability Mapping

**Figure 6:**
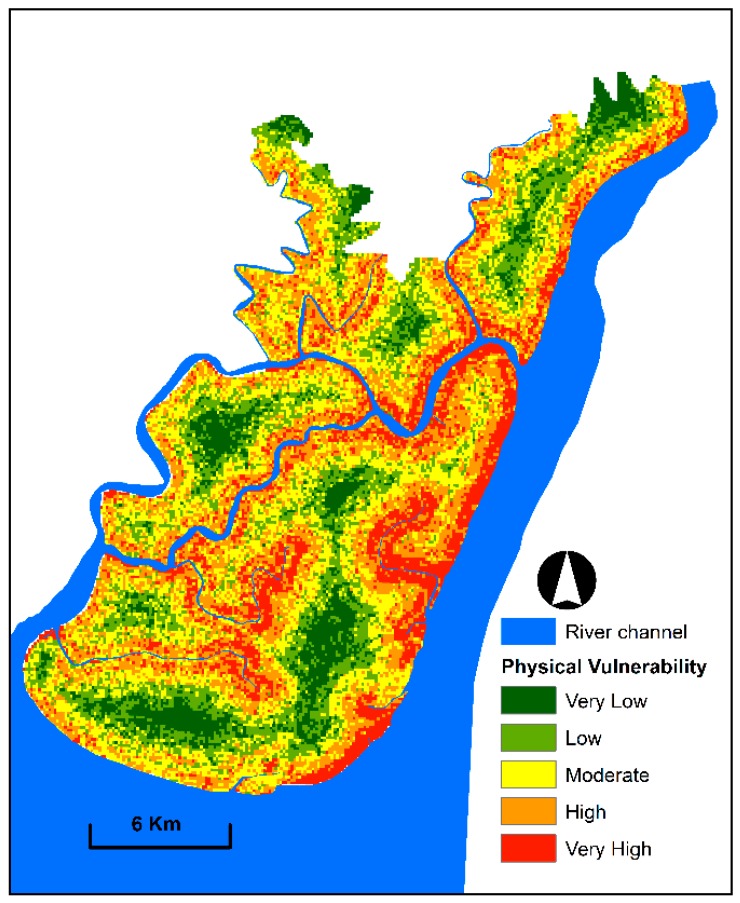
Physical vulnerability map exhibiting spatial patterns and degree of physical vulnerability to floods.

A map of physical vulnerability to floods was produced and categorised into five classes (Figure 6). The produced map demonstrated that approximately 80% of the study area was classified into moderate to very high vulnerability, whereas very low and low vulnerability covered 20%. The south-eastern, eastern and central parts and areas near the active channels are highly vulnerable to flood effects because they are close to the river channels and exhibit low elevation and gentle slope. By contrast, northern and north-western parts and few areas from the central portion of the study area are less vulnerable because they are located within high elevation, in steep slope and far from the active river channel.

### 3.2. Social Vulnerability Mapping

**Figure 7:**
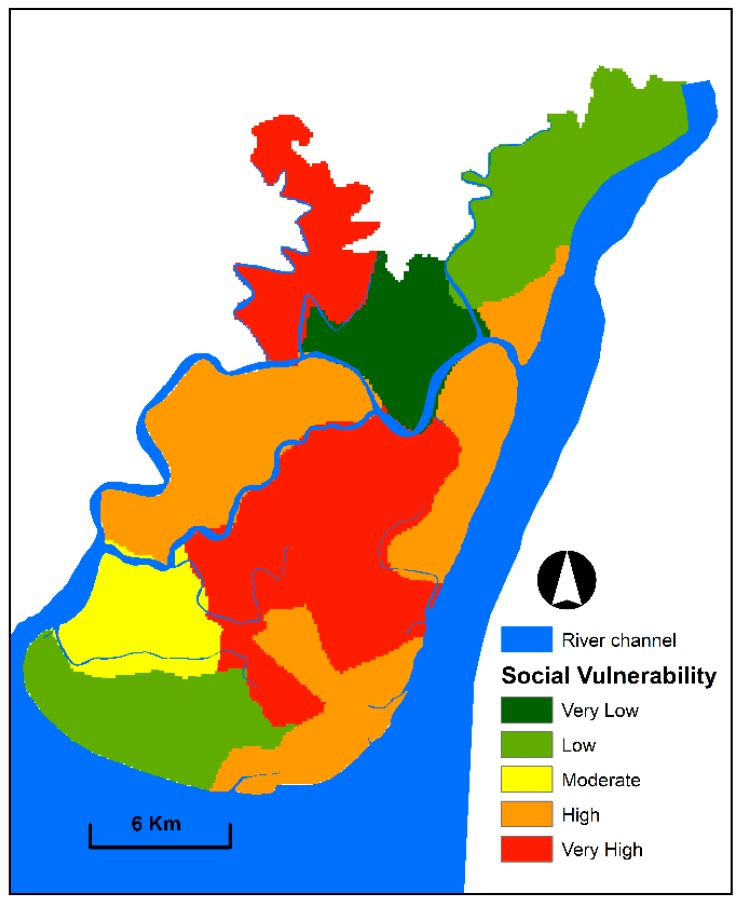
Social vulnerability map exhibiting spatial patterns and the degree of social vulnerability to floods.

Several criteria were selected to assess the social vulnerability of communities to floods. A social vulnerability index was generated from the processed criteria. The produced social vulnerability index values were categorised into five levels for creating a social vulnerability map (Figure 7). The resulting map indicates that communities living in the eastern, south-eastern and middle parts of the study area are in high and very high vulnerable zones. The high and very high socially vulnerable zones cover 34% and 24% of the total area, respectively. The social vulnerability of these highly vulnerable zones is due to the high level of population density, dependent population, unsafe sanitation systems and agriculture-dependent population. By contrast, the low and very low socially vulnerable areas cover 32%. These areas comprise the northern and south-western portions of the study area. In addition, the socio-economic condition of the communities in these areas is good.

### 3.3. Coping Capacity Mapping

**Figure 8:**
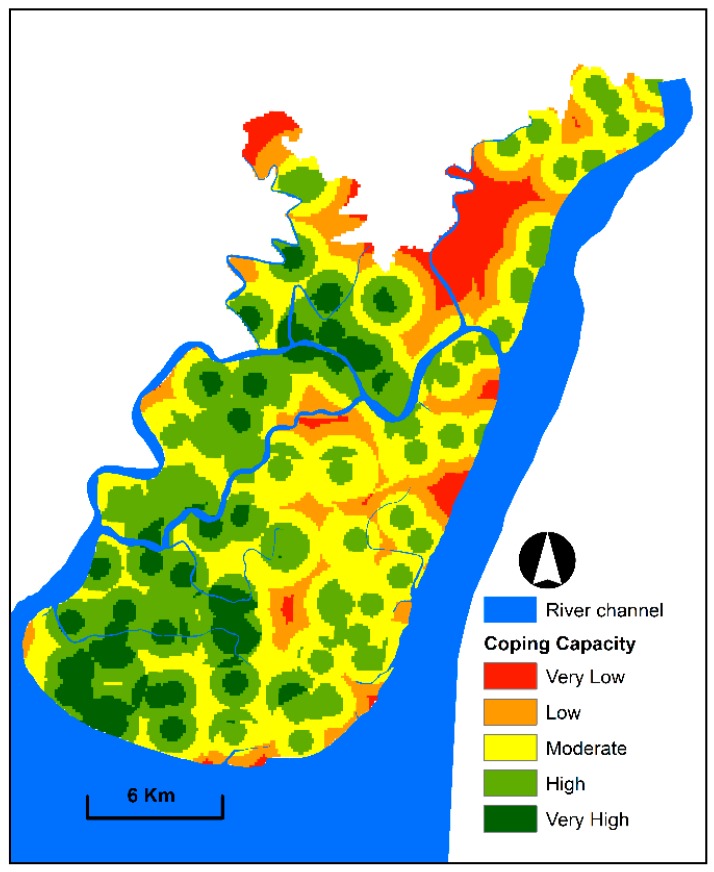
Coping capacity map exhibiting spatial patterns and the degree of coping capacity against floods.

The coping capacity map was created by categorising developed index values into five levels. Figure 8 presents that moderate to very high coping capacity levels cover 76% of the study area. People in these areas have good access to flood shelter and health complexes and are educated. An educated society can effectively cope with flood vulnerability because these people know the measures that they need to take before, during and after flood events. The coping capacity of this area is higher than that of other parts of Bangladesh given that numerous flood shelters and health complexes are recently established after the devastating effects of several floods triggered by tropical cyclones and intensive precipitation. By contrast, low to very low coping capacity zones cover 24% of the study area.

### 3.4. Vulnerability without Integrated Coping Capacity

**Figure 9:**
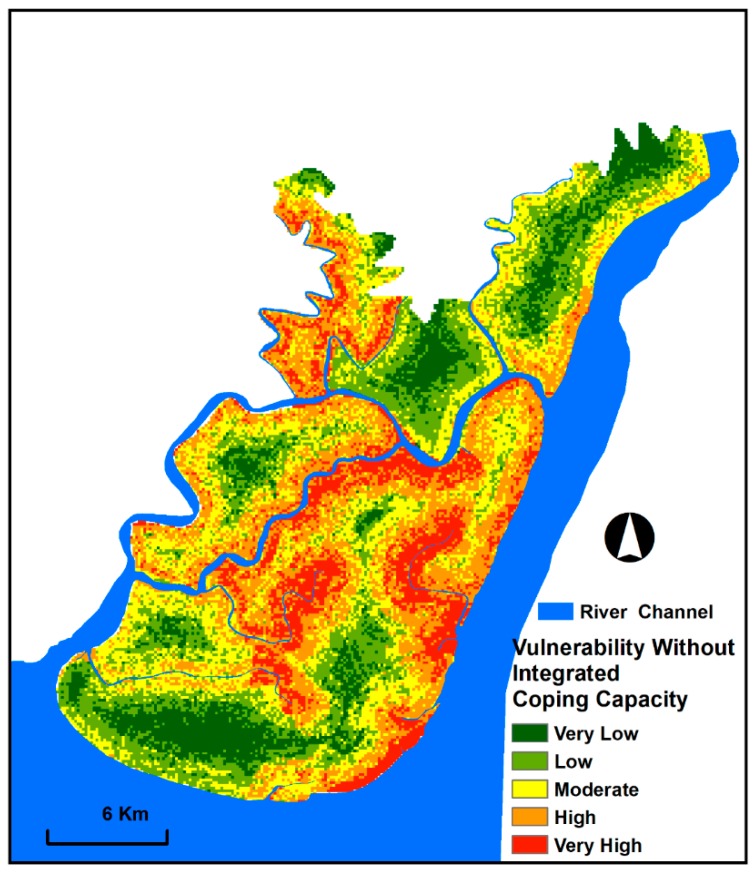
Vulnerability map without integrated coping capacity exhibiting spatial patterns and the degree of vulnerability to floods.

Vulnerability to floods without integrated coping capacity was mapped by multiplying the physical and social vulnerability indices and categorising them into five classes (Figure 9). Figure 9 presents that moderate to very high vulnerable zones account for 68% of the study area. These zones cover the south-eastern, eastern and north-eastern parts of the study area due to the closeness to the active channel, low elevation and gentle slope, high precipitation and population density, further dependent population, poor sanitation and low housing quality. By contrast, the south-western and north-eastern portions of the study area are situated in low and very low vulnerable zones and cover 11% and 21% of the land, respectively. Most of these areas are far from active channels and steep slope and exhibit moderate elevation and good socio-economic condition.

### 3.5. Vulnerability with Integrated Coping Capacity

**Figure 10:**
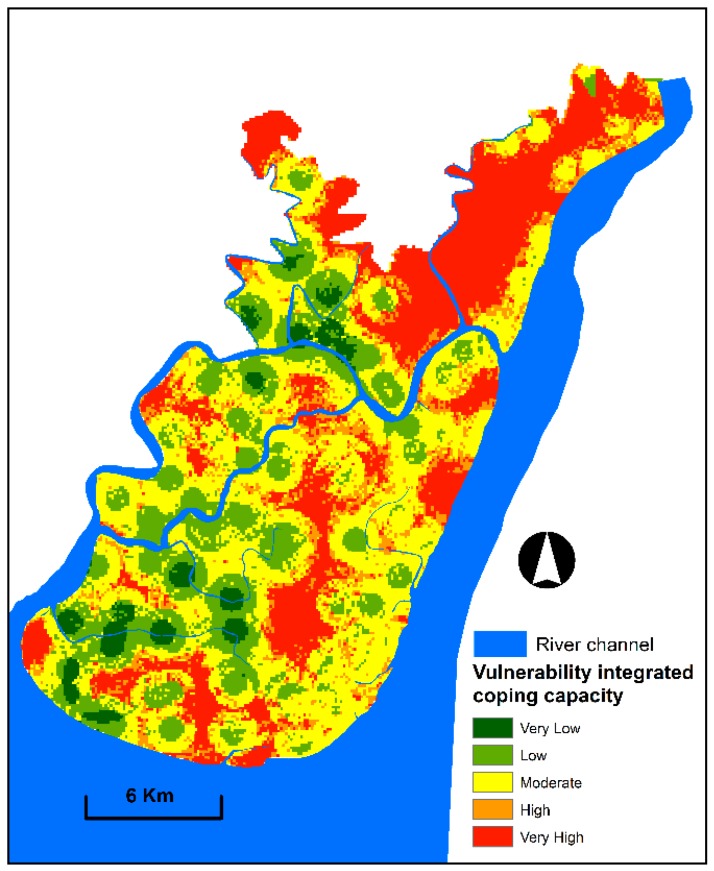
Vulnerability integrated coping capacity map exhibiting spatial patterns and the degree of vulnerability to floods.

A coping capacity-integrated vulnerability index was created by multiplying the physical and social vulnerability indices and then dividing them using the coping capacity indices. Afterwards, the coping capacity-integrated vulnerability index was categorised into five levels to produce the map (Figure 10). The produced map exhibited different results from the vulnerability map without integrated coping capacity. The areas (south-eastern and north-eastern) had very high and high vulnerability in the map without coping capacity integration. These areas are now moderate to very low vulnerable zones. Thus, incorporating the coping capacity is crucial to derive the real vulnerability scenario. By contrast, the areas in the north-eastern, north-western and central parts and lower portion of south-western part are highly vulnerable due to the low coping capacity. Figure 10 exhibits that moderate to very high vulnerable zones cover 64% of the study area, whereas very low and low vulnerability zones account for 36%.

### 3.6. Validation of Vulnerability Assessment

The adopted qualitative approach was able to provide reliable information for evaluating our spatial vulnerability assessment results. The qualitative approach covered in-depth personal observation and discussion with the local people, experts and policymakers for their opinion on the produced vulnerability maps. Our spatial vulnerability assessment results were promising based on the opinions of the local people, experts and policymakers (Table 5). Out of 60 respondents about 37 (62%) respondents were highly satisfied, 14 (23%) respondents were satisfied and 9 (15%) respondents were not satisfied with the results. Moreover, the vulnerability map without integrated coping capacity revealed that south-eastern and eastern areas are located within high to very high vulnerable zones. The field observation data obtained by the authors showed similar results. Highly vulnerable areas are affected >4 times by floods in a year. Very low and low vulnerability areas were unaffected by floods in the last 2 years. These areas are often affected once to twice a year.

## 4. Conclusions

This study presents a multi-criteria-incorporated approach of spatial flood vulnerability mapping using remote sensing, spatial analysis and field data at a local scale. Geospatial techniques were used to map all selected criteria under each component of vulnerability. An AHP was adopted in the ArcGIS environment to integrate multi-criteria in a spatial decision-making process. Kalapara Upazila, a local administrative area in Bangladesh, was used for examining the suitability of this developed approach. The produced vulnerability maps were validated through a qualitative validation approach that included in-depth personal observation and discussion with the local people, experts and policymakers in the study area to obtain their feedback on the created vulnerability maps. This study presented an efficient way for assessing the spatial vulnerability of flood effects by integrating multi-criteria using geospatial techniques at a local scale.

A local-scale study integrated with multi-criteria evaluation is required to derive the accurate and detailed vulnerability information. However, collecting spatial data at the local scale and processing and integrating them for the spatial decision-making process in data-poor countries are highly challenging. Our developed geospatial approach exhibited efficiency in generating detailed and accurate vulnerability information through multi-criteria evaluation at the local level. The AHP was useful for weighting the selected multi-criteria and spatial decision-making process. In addition, mapping actual vulnerability information requires integrating the coping capacity of the area in the vulnerability assessment process. Results showed that vulnerability was greatly influenced when coping capacity was incorporated. Furthermore, validation of the results by providing reliable vulnerable information enhanced the applicability of this approach. This study presented a framework for the overall spatial flood vulnerability assessment that integrates physical and social vulnerabilities and coping capacity. The generated information from this study could be applied by planners and administrators to develop effective flood effect mitigation strategies.

The outcomes of this study were accompanied by a number of drawbacks. Numerous criteria are required to process and map effective vulnerability assessment. Collecting quality and up to date spatial data for each criterion at the local level is highly challenging, especially in developing countries. This study addressed similar challenges. SRTM 1 DEM at 30 m spatial resolution was used to create the elevation and slope map. High spatial resolution topographic data, such as LIDAR, could provide excellent outputs. Freely available Landsat OLI imagery at 30 m spatial resolution was used for mapping land use and cover. However, high spatial resolution satellite imagery could provide excellent results. Land use and cover classification accuracy was also performed by acquiring reference points from Google Earth image instead of the field due to lack of funding and short timeframe. Our study used the most recent population census data to map social vulnerability criteria which was conducted in 2011. Up to date socio-economic data could provide better outputs. Furthermore, our results were validated by qualitative judgment. Quantitative judgment can effectively justify the developed approach. Future studies can address the listed drawbacks. The developed approach is still considered useful for mapping spatial vulnerability at the local scale to support flood management initiatives in spite of the drawbacks. This verified approach can be applied in other similar environments for mapping spatial flood vulnerability by modifying the criteria, data type and scale if necessary.

## Figures and Tables

**Table 1:** Data type and sources used in this study.

Data Type	Source	Period	Mapping Output
Landsat 8 OLI	United States Geological Survey (USGS) Earth Explorer	20-01-2018	Land use and cover
SRTM-DEM (30 m resolution)	USGS Earth Explorer	11-2-2000	Elevation and slope
River channel	USGS Earth Explorer	20-01-2018	Distance to active channel
Precipitation	Bangladesh Meteorological Department (BMD)	2004–2014	Precipitation
Population	Bangladesh Bureau of Statistics (BBS)	Population census of 2011	Population density, dependent population, female population, literacy rate
Wooden house	BBS	Population census of 2011	Wooden house
Household with pond and others	BBS	Population census of 2011	Household with pond and others
Household with no sanitation	BBS	Population census of 2011	Household with no sanitation
Flood shelter and health complex	Fieldwork	August–October	Distance to flood shelter and health complex

**Table 2:** Alternative ranking scheme based on the contribution to risk flood disaster.

Component	Criteria	Ranking (Based on Vulnerability)				
		Very Low (1)	Low (2)	Moderate (3)	High (4)	Very High (5)
**Physical vulnerability**	Elevation (m)	>10	7–10	5–7	2–5	<2
	Slope (%)	>6.56	3.74–6.56	2.14–3.74	0.93–2.14	<0.93
	Precipitation (mm/year)	<7.1	7.1–7.2	7.2–7.3	7.3–7.4	>7.4
	Land use and cover	Open water bodies	Bare land	Vegetation	Cropland	Settlement
	Distance to active channel (m)	>2400	1800–2400	1200–1800	600–1200	<600
**Social vulnerability**	Population density (km^2^)	<360	360–400	400–450	450–500	>500
	Dependent population (%)	<46	46–47	47–48	48–49	>49
	Disabled population (%)	<1	1–1.5	1.5–2	2–2.5	>2.5
	Female population (%)	44.5	44.5–48.5	48.59–49.7	49.7–50.4	>50.4
	Wooden house (%)	74.0	74.0–83.7	83.7–86.6	86.6–89.8	>89.8
	Household with ponds and others (%)	<20	20–30	30–40	40–50	>50
	Household with no sanitation (%)	<10	10–15	15–20	20–25	>25
	Agriculture-dependent population (%)	3.15	3.14–4.68	4.68–6.28	6.28–8.92	>8.92
**Coping capacity**	Literacy rate (%)	>55	50–55	45–50	40–45	<40
	Distance to flood shelter (m)	<600	600–1200	1200–1800	1800–2400	>2400
	Distance to health complex (km)	<2	2–4	4–6	6–8	>8

**Table 3:** Scale of relative importance (adapted from Saaty [47]).

Relative Importance	Definition	Description
1	Equal importance	Two factors equally influence the objective
3	Moderate importance	Experience and judgment slightly favour one factor over another
5	Strong importance	Experience and judgment strongly favour one factor over another
7	Very strong importance	One decision factor is strongly favoured over another, and its supremacy is established in practice
9	Extreme importance	The evidence favouring one decision factor over another is of the highest possible orders of validity
2, 4, 6 and 8	Intermediate values between adjacent judgement	When compromise is required

**Table 4:** Weighting the criteria using AHP.

Component	Criteria	Weight
Physical vulnerability	Elevation	0.22
	Slope	0.16
	LULC	0.08
	Precipitation	0.12
	Distance from active channel	0.42
CR: 0.04		
Social vulnerability	Population density	0.11
	Dependent population	0.17
	Female population	0.17
	Wooden house	0.10
	Household with ponds and others	0.05
	Household with no sanitation	0.05
	Disabled population	0.28
	Agriculture-dependent population	0.08
CR: 0.04		
Coping capacity	Literacy rate	0.12
	Number of shelter houses	0.61
	Number of hospitals	0.27
CR: 0.05		

**Table 5:** Summary of feedback on flood vulnerability results acquired from a different category of people during the field visit.

Category of People	Total Number of Respondents	Feedback		
		Highly Satisfied	Satisfied	Not Satisfied
Experts	5	3	1	1
Policymakers	5	2	2	1
General people	50	32	11	7
Total	60 (100%)	37 (62%)	14 (23%)	9 (15%)
